# Pragmatics partially segregated from Theory of Mind: evidence from resting-state functional connectivity

**DOI:** 10.1098/rstb.2023.0498

**Published:** 2025-08-14

**Authors:** Christoffer Forbes Schieche, Manu Mahal, William Hedley Thompson, Julia Uddén

**Affiliations:** ^1^ Department of Linguistics, Stockholm University, Stockholm, Sweden; ^2^ Department of Psychology, Stockholm University, Stockholm, Sweden; ^3^ Department of Applied Information Technology, University of Gothenburg, Goteborg, Sweden; ^4^ Department of Clinical Neuroscience, Karolinska Institute, Stockholm, Sweden

**Keywords:** pragmatics, Theory of Mind, functional connectivity, resting state, fMRI

## Abstract

Pragmatics and Theory of Mind (ToM) are at play during conversational interaction, but the relationship between the two is a matter of debate. Using resting-state fMRI data, we investigate a potential segregation of the two domains by considering functional connectivity within and between the ToM and language networks, and their relation to pragmatic behavioural measures. We also study the connectivity of two cortical clusters, one in the left intraparietal sulcus and one in the bilateral dorsal precuneus. These clusters are located outside both the ToM and language networks and were previously found to covary with individual pragmatic variability. The results show that these two clusters are functionally connected at rest and that their degree of connectivity is related to pragmatic behaviour. On the other hand, there was no relation between pragmatic behaviour and degrees of connectivity involving the ToM and language networks. Furthermore, the two clusters were not connected to either the ToM or language networks. In conclusion, we suggest that the domain of pragmatics is partially segregated from ToM, and provide further support that the two clusters outside the ToM and language networks are pragmatically relevant.

This article is part of the theme issue ‘At the heart of human communication: new views on the complex relationship between pragmatics and Theory of Mind’.

## 1. Introduction

To make themselves understood in conversation, interlocutors must at times rely on capabilities beyond structural language. The domain of pragmatics includes several phenomena, among them the tuning of utterances based on assumptions of a target interlocutor (i.e. audience design); understanding non-literal language, such as irony and metaphor; conversational phenomena, such as turn-taking; and speech act processing, e.g. of indirect speech acts (ISAs). Theory of Mind (ToM) is a similarly multifaceted domain which e.g. allows the ascription of mental states to others [1]. It is not controversial to assume that both pragmatics and ToM are at play during common verbal interaction. However, the relationship between pragmatics and ToM, as two canonical bundles of cognitive processes relevant to conversation, is neither settled theoretically nor empirically.

The principal aim of this study is to contribute to the understanding of the relationship between pragmatics and ToM. We test whether combined behavioural and neuroimaging measures of pragmatic individual differences are linked to the functional architecture of the brain at rest, with a particular interest in the ToM network. In this introduction, we will start by outlining and summarizing the possible relations between pragmatics and ToM, and mention related theoretical and empirical (behavioural) work on this topic. Then, we will present findings and suggestions from previous fMRI studies on pragmatics. Finally, we will describe the assumptions of the functional connectivity method we use in the current study. While our main focus is on pragmatics and ToM—the theme of this special issue—we additionally investigate the relationship between pragmatic individual differences and the language network.

### (a) Possibilities

**Figure 1.**
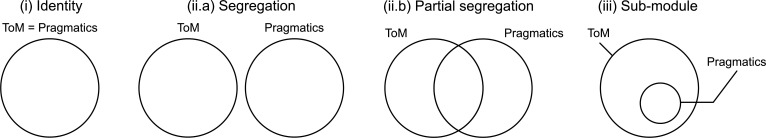
Possible relations between ToM and pragmatics.

We will start by considering the different possible relationships of the domains of ToM and pragmatics in figure 1: (i) identity, where these abilities are entirely one and the same, or at least use the same underlying processes, but in different circumstances; (ii) segregation, where it makes sense to model two separate domains (see ii.a), though there may be degrees of overlap (as in a partial segregation, see ii.b) where they e.g. share common subprocesses or contribute to each other in a highly integrated manner. Empirically, it is essentially the model of a *partial* segregation that is feasible to test, as a complete segregation would only be shown if any potential overlap could be excluded. We also regard the degree of segregation as meaningful to discuss in this context. (iii) Finally, Sperber & Wilson [2] have previously theorized that ToM is a domain encompassing pragmatics. The opposite relation of pragmatics encompassing ToM can be disregarded as there are aspects of ToM far removed from language and conversation [3].

### (b) Positions

Present theoretical accounts in the literature favour either (ii) or (iii) out of the possible configurations. Andrés-Roqueta & Katsos [4] propose a segregational account, where aspects of pragmatics that do not require ToM (called *linguistic-pragmatics*) are distinguished from those that do (*social-pragmatics*). They further argue for a situation-based view of pragmatics and ToM (or ‘mind-reading’), where it is the communicative situation that determines whether ToM is necessary (or not), rather than the specific pragmatic phenomenon [5]. Accordingly, the degree of segregation is fluid and dependent on context. This stands in contrast to other segregational accounts that attempt to classify certain pragmatic phenomena as needing ToM or not, irrespective of context [6].

As already indicated, the configuration in (iii) has been proposed by Sperber & Wilson [2], which places pragmatics within a larger construct of ToM. We do not refer to this view as segregational, as pragmatics is considered a sub-module of ToM, specialized for *comprehension*. While both (ii) and (iii) distinguish between ToM and pragmatics, the latter view’s inclusion of pragmatics in the larger scope of ToM has been criticized and the available data (see §1c) seem to at least partially segregate the two. Indeed, *linguistic-pragmatics* [4] include phenomena that become a problem for the sub-module view, as those phenomena are pragmatic but argued to occur without ToM.

### (c) Empirical evidence from behavioural studies

A general problem for empirical tests of these views is that experimental paradigms are not precise in distinguishing what they are testing (ToM or pragmatics [7]) and seldom test ToM and pragmatics simultaneously. Nonetheless, as we will now review in more detail, empirical data in both neurotypical and atypical populations are informative to some extent. In summary, behavioural studies on autism spectrum disorder (ASD), developmental populations, as well as adult individual differences, have found mixed results as to how pragmatics and ToM relate, as well as on whether our structural language and pragmatic competences are associated.

#### (i) Studies on autism spectrum disorder

Deficits in ToM have been considered as seemingly universal within the ASD population [8]. Similarly, pragmatic deficits are ubiquitous [9]. However, in Kissine’s [10] review of several studies, regarding the performance of individuals with ASD compared with typically developing (TD) peers in pragmatic tasks, there are some cases where ASD individuals perform similarly to TDs. The suggestion is that they could achieve this by other means that do not necessitate ToM, e.g. that the linguistic content alone is sufficient for successful conversational inferences. However, such inferences are not necessarily drawn within the language network itself, but may instead draw on other cognitive resources. Furthermore, Andrés-Roqueta & Katsos [11] showed that in children with ASD or developmental language disorder and language-matched controls, performance in a linguistic-pragmatic task was associated with structural language skill, and a social-pragmatic task with both language and ToM, where the ASD participants showed impaired performance. Hence, while ToM competence sometimes affects pragmatic outcomes, this literature suggests that there are cases where ToM has no such effect. This would support a partial segregation of ToM and pragmatics.

#### (ii) Studies on typical development (children/adolescents)

In the TD population, Arvidsson *et al.* [12] investigated the development of audience design in adolescents (ages 11–12 and 15–16), and showed that the younger group did not adapt utterances based on an interlocutor’s presumed world knowledge (based on age) to the same extent as their older peers. However, the different age groups did not differ when (subsequently) asked to judge whether they believed an interlocutor knew specific targets (i.e. attributing knowledge through ToM). In other words, the two adolescent groups had similar capabilities of knowledge attribution outside of the pragmatic production context, but the younger group did not use this capability to the same extent, at least not in the task context. This suggests some partial developmental segregation of ToM and pragmatics, where the development of related aspects of ToM and pragmatics occur in the named order. If this pattern generalizes, one may take a perspective on the pragmatic (albeit versatile) domain as a context-specific (e.g. to the conversational context) expert domain, building on ToM, while at the same time being partially segregated from it (see further discussion in §4c).

#### (iii) Studies on individual differences in the adult typical population

Wilson & Bishop [13] studied the potential segregation of pragmatic and structural language skills by analysing patterns of individual differences in a large cohort of TD adults. By modelling a large test battery completed by 120 participants, they obtained results indicating that pragmatics and language skills are at least partially segregable, as a two-factor model dividing the two had significantly better fit than a one-factor model. In our previous study, Bendtz *et al.* [14], we used a similar design to show a partial segregation of pragmatics and ToM in a sample of 199 young adults. Neither of two behavioural pragmatic tests on prosodic comprehension (PC-RR, see §2a(i)) or audience design (AD, see §2a(ii)), correlated with a standard test of non-verbal ToM (reading the mind in the eyes-task, RMET), again supporting the notion of partial segregation. This previous study also contained an fMRI experiment on a subset of the participants (*n* = 61) at a later date. When testing vocabulary skill (a part of structural language) in these participants, the test score correlated with both pragmatic test scores (0.32 and 0.44, respectively), indicating a somewhat stronger relation than the corresponding correlation in Wilson & Bishop (0.2, vocabulary versus implied meaning [13]. We will return to the fMRI part of this experiment shortly.

### (d) The neuroimaging approach

As a complement to behavioural testing of the relationship between ToM and pragmatics, we suggest neuroimaging to be a promising methodological approach, e.g. somewhat less dependent on the details of task design. By measuring brain activity during pragmatic tasks or functional connectivity at rest, we can observe how pragmatic processing manifests in the human brain, and to what extent there are overlaps or covariation with other regions known to subserve ToM (i.e. the ToM network, cf. [3]).

#### (i) Pragmatics

As the domain of pragmatics includes multiple phenomena, one cannot test or make claims of the entirety of this domain from a single experiment. In the experimental design of our previous fMRI study [14], we focused on a pragmatic phenomenon that requires making pragmatic inferences that rely on the conversational context, i.e. indirect speech acts (ISAs). Here, the meaning of a complete utterance (as opposed to specific words; see below) must typically be inferred considering the previous conversational context. A number of fMRI studies contrasting direct speech acts and ISAs of a similar sort as our previous study, have consistently shown more activation during the latter, in regions related to ToM and language, both in adults and adolescents [15–17]. This has been suggested to mean that making pragmatic inferences (in this case, understanding what someone means beyond the literal utterance) increases the demand of both structural language and ToM systems. However, a recent study by Feng *et al.* [18] also included pragmatic inferences driven by specific words (such as scalar expressions like ‘some’ versus ‘all’) rather than complete utterances (as in the other studies). Intriguingly, these types of inferences did not recruit ToM, though activation within the language network was similar in both kinds of inferences.

As we have now reviewed fMRI experiments that have used ISAs to operationalize pragmatics, the next step is to note that the general findings of the ISA experiments (including Bendtz *et al*. [14]) seem to generalize to other pragmatic phenomena. Hauptman *et al.* [19] performed a meta-analysis on fMRI studies covering ten types of non-literal language comprehension tasks (including ISAs in this category), resulting in six clusters in the left hemisphere. The majority of these clusters covered areas within the ToM and language networks. Four of the clusters show some overlap with activations found in Bendtz *et al*.’s basic ISA-contrast over all participants. More specifically, the overlap was observed in ToM and language-related regions in the left anterior STG/STS, left IFG, left TPJ (cluster 5 in [19]) and the left dorsal MPFC (other authors have called this area the medial SFG [20]).

In the fMRI experiment of Bendtz *et al.* [14], we also asked whether there was individual pragmatic variability in neural activity (while making pragmatic inferences), and if variability in behavioural and neural signatures of pragmatics could be explained by variability measured behaviourally in structural language skills, executive functions or ToM. As already mentioned, to capture individual variability, we administered a battery of behavioural tests (healthy participants, *n* = 199). Two of these behavioural tests were specifically designed to cover individual differences in pragmatic competence in a broad sense, spanning production (AD) and comprehension tasks (PC-RR), the former being a verbal task and the latter using prosodic features, hence going in the non-verbal direction. These tests are further described in the method section (§2a(ii), a(i)). In addition to using these tests in order to investigate the covariation of ToM and pragmatics reported above, we also used the test scores from these pragmatic tests to form two groups, one with low scores (LS) on both tests, which were compared with those with high scores (HS). These groups were further investigated with the ISA task in the scanner, and they are also used in the current study on resting-state fMRI data belonging to the same participants.

#### (ii) fMRI task (indirect speech acts) results and the need for resting-state investigation

In our previous study, we used an adapted version of the established ISA experiment [14]. We played short recordings of dialogues, with an introductory context followed by a question and a reply. The recorded contexts and questions varied throughout the trials, whereas replies with identical linguistic form were rendered either direct or indirect by the varied preceding context and question. The indirect replies were aimed at prompting participants to make pragmatic inferences, and the main analysis contrasted these with the direct replies.

The basic results replicated previous studies using ISA paradigms. Both groups showed activity in the ToM and language networks. More interestingly, in our unique group comparison of high versus low pragmatic score participants, we showed activation in two posterior clusters outside language and mainly outside ToM regions. These regions were also principally not among those found in the previously mentioned meta-analysis by Hauptman *et al.* [19]. More precisely, regions more activated for the HS group during ISA (interaction HS > LS, indirect > direct) were: (i) the left superior and inferior lateral parietal cortex, centered in the anterior/mid intraparietal sulcus, and (ii) the bilateral dorsal precuneus (henceforth, collectively called the ParPrec-clusters). Additionally, there were no significant correlations between either of the two clusters’ average signal intensity in the indirect > direct contrast and the behavioural scores of ToM or language (see electronic supplementary material, figure S1).

We hence concluded that pragmatic processes can be *partially* segregated from structural language and ToM processes. As for the segregation of pragmatics and ToM, this builds on three observations: (i) a minimal overlap of the ISA-relevant ParPrec-clusters with ToM areas (the dorsal precuneal cluster shared only 1% of voxels with a meta-analytic mask of ToM from Neurosynth [21]); (ii) absence of correlation between pragmatic and ToM (RMET) behavioural tasks (*n* = 199); and (iii) absence of correlation between the ISA-relevant ParPrec-clusters and behavioural ToM (RMET) results. We suggested that the results in the indirect > direct contrast reflect low-level pragmatics, whereas the interaction results (i.e. the ParPrec-clusters), reflect higher levels of pragmatic processing (see further [22]). By design, the described interaction is related to making pragmatic inferences. As the group division itself was based on pragmatic measures (AD and prosodic speech act comprehension), these can also be considered, at least indirectly, implicated. However, what was not clear from this part of the design was whether the activity in the ParPrec-clusters reflects a joint or two separate (pragmatic) processes.

In the present study, we further investigate whether ToM can be partially segregated from pragmatics. Before performing the pragmatic ISA task in the scanner in our previous study, we collected data from the participants at rest (so-called resting-state fMRI). Using this data, we now study different regions’ co-activation (i.e. functional connectivity) and whether the two groups, differing in pragmatic skill level, have different degrees of functional connectivity in relevant canonical or predetermined networks. The first aim is thus to see whether the ParPrec-clusters represent two different or one joint process. If the clusters are functionally linked during rest (potentially, at least for skilled pragmatic participants), this would be an indication that the activation observed in Bendtz *et al.* [14] reflects a joint process. We also test whether the level of connectivity within these pragmatically related clusters differs significantly between the groups that were the basis of the initial finding during the ISA paradigm in Bendtz *et al. *In addition, we investigate functional connectivity and potential group differences within the ToM network, between ToM and the language network (ToM–Lang), and between ToM and the ParPrec-clusters (ToM–ParPrec). Paunov *et al.* [23] have previously investigated both within-network ToM and between-network ToM–Lang connectivity, and showed above baseline connectivity at rest. Beyond replication, we have added the possibility of testing for group differences based on pragmatic behavioural results. Our novel connectivity comparison (ToM–ParPrec) additionally allows us to test whether the ParPrec-clusters are functionally linked to the overall ToM network at rest. If not, this would again suggest a larger degree of partial segregation.

In addition, in a final separate line of inquiry, we test whether there are group differences in functional connectivity within the language network (Lang) and between the language network and the ParPrec-clusters (Lang–ParPrec). This is to further delineate the functional role of the ParPrec-clusters vis-à-vis the language network, as well as testing for partial segregation of pragmatic aspects from language, operationalized as functional connectivity within the language network.

#### (iii) Functional networks

To briefly expand on the basis of the method used in this study: human brain function is partly realized by interactions between dispersed regions [24], interacting in functional networks. Interconnected regions are considered to share information with one another [24,25]. A number of large-scale brain networks have been defined since the 1990s [26,27], supporting a variety of functions, such as ToM and language. We revisit neuroimaging studies of pragmatics in the context of such large-scale networks in the discussion (§4d).

The current study capitalizes on the finding that functional networks consist of regions showing spontaneous and correlated activity, also during rest [28,29], established using resting-state fMRI. Such correlated activity is particularly observed in low frequency oscillations (approx. 0.01−0.1 Hz). Additionally, networks that consistently activate during particular tasks also show correlated activity during rest. This includes correlated activity (i) within regions of the ToM network, (ii) within regions of the language network and (iii) between the two networks [23]. However, Paunov *et al.* [23] had no behavioural measurements, so whether individual variation in connectivity within and between the included regions can be correlated to behavioural outcomes is not clear. Here is where the design and participant sample from our previous study [14] may help, as we acquired both resting-state data and measures of individual differences in pragmatic skill.

### (e) Summary of the current study

In summary, we here investigate the relationship between pragmatics and ToM. This study exclusively makes use of data acquired in our previous investigation [14]. This includes (as-of-yet not analysed) resting-state data and we use the same group division (HS and LS) based on participants’ behavioural data on pragmatic skills.

If there are no group differences in functional connectivity within the ToM network, this would be evidence for partial segregation between pragmatics and ToM. Similarly, the absence of group differences in the between-network comparisons including ToM (ToM–Lang and ToM–ParPrec) would support the same conclusion. We are also interested in the functional role of the ParPrec-clusters themselves, manifesting in tests of group differences in within-network connectivity of these clusters, as well as a separate parallel investigation of group differences in Lang–ParPrec connectivity. Finally, as structural language function itself can be operationalized as functional connectivity in the language network, we are also interested in whether our group differences in pragmatic ability covary with such connectivity. If not, that would be evidence for relative independence of the three pragmatic tasks relevant to this study (AD, PC-RR and ISA) from the structural language domain.

## 2. Methods

### (a) Participants

Our previous investigation, Bendtz *et al.* [14], consisted of two experimental sessions. First, we obtained behavioural data in a battery of tests from 199 participants (99 males; mean age 28.7 (males) and 29.3 (females)). The battery included two pragmatic tests that were designed specifically for the study: prosodic comprehension of request for response (PC-RR) and audience design (AD), targeting pragmatic comprehension and production, respectively (see §2a(i),a(ii) and [14]). We designed new tests to ensure sufficient sensitivity to capture individual variation in an adult population. Several months after the first session, 61 participants (age 18−36, 28 males) from the behavioural sample were invited to participate in a subsequent fMRI experiment, including resting-state (as-of-yet not analysed, but the basis for the current study) and task runs (analysed in [14]). Participants were chosen for the fMRI experiment based on having among the 50% best scores on both tasks for a high score (HS) group, or 50% worst scores on both tasks for a low score (LS) group. Additional inclusion and exclusion criteria for both groups were that participants had to be native Swedish speakers, having no history of neurological impairment, brain surgery, ASD diagnosis or language impairment. Each participant was given 350 SEK post-experiment. The study was approved by the Regional Ethical Review Authority in Stockholm. Two out of the 61 participants were removed before the current analysis: one due to ending participation before completion and one due to unclear group membership. The final sample of 59 participants was divided between the groups HS = 28 (mean age 30.5) and LS = 31 (mean age 29.2).

#### (i) Prosodic comprehension of request for response

We designed the PC-RR test to probe the participants’ skill in evaluating whether the utterance of a speaker was intended as a statement or a request for feedback. Hellbernd & Sammler [30] reported above chance level correct identification of speech acts based on the prosody of single words (including pseudo-words). Prosody can thus be regarded as another means to modify and interpret the intended meaning of utterances, beyond contextual information derived from semantics or syntax. Our test consisted of twelve sentences, each recorded in two conditions. In the control condition, each sentence was spoken with prosody indicating a statement, i.e. *not* requesting feedback. In the test condition, the same sentence was produced with a modulated prosody to indicate uncertainty, i.e. requesting feedback. No participant was presented with the same sentences across conditions. Each participant was presented with the twelve sentences in a randomized order, evenly split between the conditions, and classified each utterance as belonging to either category by pressing a button.

#### (ii) Audience design

We also designed a version of the director’s task (DT) [31] to test pragmatic production. There have been criticisms aimed at the DT that it may rather be testing selective attention than pragmatic production [32], as well as other aspects of task validity [33]. In the standard DT, participants label visually presented objects in a bookshelf as unique to their point of view, or as being visible to a listener on the other side of the bookshelf. This is regarded as a manipulation of common ground. In our modified version of the DT, we realized the manipulation of common ground in terms of word knowledge, rather than visual perspective. We suggest that real-life examples of compromised common ground, relevant for conversation, more often come in terms of word knowledge, as compared to visual perspective, and that the task thus increases ecological validity. We therefore varied the age, gender and cultural background of an imagined listener, so that the participant would need to tailor their utterance according to their assumption about a specific listener’s knowledge state. The pragmatic behaviour that the AD test targets is the successful online usage of these assumed knowledge states when tailoring one’s utterance, although ascribing different knowledge states to others can be considered to necessitate ToM. In each trial, participants were presented with an image of a bookshelf with five objects: one target object, one competing object and three filler objects. Participants were asked to label a target object in the bookshelf that could be assumed to be unknown (test condition) or known (control condition) to a specific addressee. Successful labelling in the unknown condition would be a description of the target in words other than its name, so that the imagined listener would have been able to correctly identify the target, as well as differentiate it from a competing object sharing some qualities with the target (e.g. a drone versus a helicopter).

### (b) General approach/procedure

The current experiment was made up of two parts: the pilot study (*n* = 8) and the full study (*n* = 51). The participants in the pilot study were randomly chosen, though equally divided between the two groups, HS and LS. We used our pilot study to formulate and narrow down hypotheses for the full study. As both partitions were derived from the same dataset (i.e. [14]), we combined the separate pilot and full study results in a meta-analytical Bayes factor as a final step (see §2d(i)).

#### (i) Regions of interest

**Figure 2.**
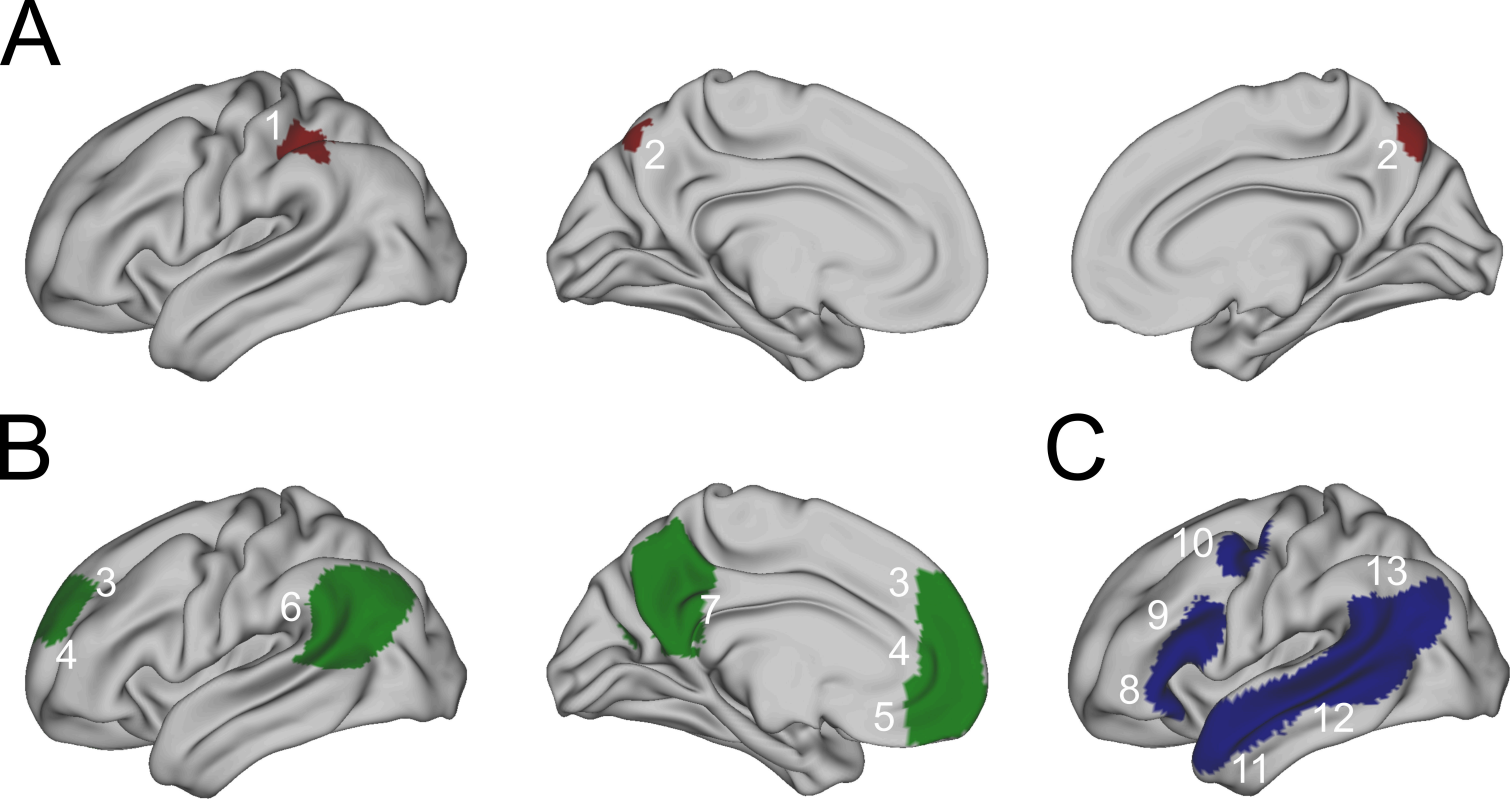
Regions of interest (ROIs) included in the analysis. (A) ParPrec-clusters from Bendtz *et al.* [14]: 1. left intraparietal sulcus (Par), 2. bilateral dorsal precuneus (Prec). (B) Theory of Mind ROIs from Dufour *et al.* [34], all in the left hemisphere: 3. dorsomedial prefrontal cortex, 4. middle medial prefrontal cortex, 5. ventromedial prefrontal cortex, 6. temporo-parietal junction, 7. posterior cingulate cortex and ventral precuneus. (C) Language ROIs from Fedorenko *et al.* [35], all in the left hemisphere: 8. inferior frontal gyrus orbital part, 9. inferior frontal gyrus, 10. middle frontal gyrus, 11. anterior temporal cortex, 12. posterior temporal cortex, 13. angular gyrus.

The locations of anatomical regions of interest (ROI)/clusters used in the analyses, i.e. the ParPrec-clusters and the ToM and language networks, are visualized in figure 2.

The ParPrec-clusters, i.e. the two clusters found in Bendtz *et al.* [14] interacting with pragmatic skill, were (i) the left anterior/mid intraparietal sulcus and (ii) the bilateral dorsal precuneus. These regions are visualized in figure 2A. As reported by Bendtz *et al*., these regions lay outside of both the language and ToM networks (1% of voxels in the precuneal cluster overlapped with the ToM network, when compared with masks from Neurosynth [21]). However, approximately 12% of the voxels in the precuneal cluster overlapped with a ToM ROI (see below) in the left posterior cingulate cortex/ventral precuneus.

We limited our main analysis to regions located in the left hemisphere regarding the ToM and language networks (see electronic supplementary material, S2.1, for our rationale), as we expected that limiting our analysis to only the left hemisphere would not produce significantly different results than with an analysis approach including the right homologous regions.

As Paunov *et al.* [23] have previously performed tests relevant for us regarding these networks, we chose to use the same network definitions. The ROIs corresponding to regions within the ToM network were taken from Dufour *et al.* [34] (available at http://saxelab.mit.edu/use-our-theory-mind-group-maps). These stem from 462 participants’ brain activity during a (false) belief > photo contrast. The regions included in this study were the (1) dorsomedial prefrontal cortex, (2) middle medial prefrontal cortex, (3) ventromedial prefrontal cortex, (4) the temporo-parietal junction, and (5) the posterior cingulate cortex and ventral precuneus. These five regions correspond to 3—7 in figure 2B.

The ROIs for the language network were based on 220 participants’ activity while being exposed to a sentence > non-words contrast (available at http://web.mit.edu/evlab/funcloc/). These ROIs are updates of those originally reported in Fedorenko *et al.* [35] with 25 participants. The included regions were the (1) inferior frontal gyrus, orbital part, (2) inferior frontal gyrus, (3) middle frontal gyrus, (4) anterior temporal cortex, (5) posterior temporal cortex and (6) angular gyrus. These regions correspond to 8—13 in figure 2C.

#### (ii) fMRI procedure

The complete experiment was performed double-blind, i.e. neither the experimenters nor participants knew which group (HS or LS) the participants belonged to. During resting-state fMRI-data collection, participants were asked to close their eyes, lie still and relax for 7 min. Task-induced activation from subsequent task runs was avoided by collecting resting-state data as the first scan.

#### (iii) Data acquisition and preprocessing

Bendtz *et al.* [14] acquired the structural and functional resting-state data at Stockholm University Brain Imaging Centre (SUBIC). The collected data were preprocessed using the statistical parametric mapping software SPM12 (https://www.fil.ion.ucl.ac.uk/spm/) [36], in MATLAB [37]. Additional preprocessing was performed using the CONN toolbox (RRID:SCR_009550, release 22 a; [38]), using default settings. Preprocessing included normalization, smoothing and segmentation (of the structural data), motion correction, coregistration, outlier volume detection, linear detrending and denoising through temporal band-pass filtering and by regressing out potential confounding effects. A detailed description of the settings of the data acquisition and preprocessing steps can be found in electronic supplementary material, S2.2.

#### (iv) Correlation analysis

We obtained a single BOLD signal time course for each ROI, by averaging all the voxel-wise time courses throughout all voxels. The single BOLD signal time courses for each ROI were first correlated against any other ROI’s time course specified in the analysis, with Pearson’s product-moment bivariate correlation, and then Fisher-transformed to increase normality [39]. For the within-network correlations (ParPrec and ToM), each pairwise ROI-to-ROI correlation was averaged per participant and then on the group level (HS or LS). The same procedure was followed for the between-network correlations (ToM–Lang and ToM–ParPrec), where either ROI of a pairwise correlation belonged to different networks. The ParPrec comparison included one pairwise correlation; ToM 10 correlations (within-network calculation (5 ROIs × (5 − 1) ROIs)/2)); ToM–Lang 30 correlations (between-network calculation, 5 × 6 ROIs) and ToM–ParPrec 10 correlations (5 × 2 ROIs). We added two additional analyses during the review process (see §2e): one within-network correlation in the language network (Lang) and one between-network correlation between the language network and the ParPrec-clusters (Lang–ParPrec). The Lang comparison consisted of 15 correlations (6 ROIs × (6 – 1) ROIs/2), and the Lang–ParPrec comparison 12 correlations (6 × 2 ROIs).

### (c) Pilot study and derived hypotheses, power analysis and preregistration (ParPrec, ToM, ToM–Lang and ToM–ParPrec)

We ran a pilot study to (i) formulate and narrow down hypotheses for the full study and (ii) specify the direction and type (i.e. one-tailed or two-tailed) of subsequent *t*-tests for group differences, based on the hypotheses. The pilot sample consisted of eight randomly selected participants (four from each group), out of the 59 participants. We performed four two-sample, two-tailed tests for group differences of functional connectivity between and within regions/networks (see §3a for a full report). One of the tests in the pilot showed support (i.e. nominal significance, see second paragraph in §2d) for a group difference: higher connectivity between the ParPrec-clusters for the HS group. We hypothesized that this result would hold in the full sample. None of the other tests rendered significant results or Bayesian support for group differences, which we similarly hypothesized to hold in our further analysis. While we based the settings of the subsequent *t*-tests in the full sample on the results from the pilot, no changes were made to the preprocessing or analysis pipelines after running the pilot study.

We performed a power analysis using the effect size from the ParPrec-connectivity comparison to calculate the desired sample size. With Cohen’s *d* = −1.987, alpha = 0.05 and power = 0.8, the projected sample size needed per group was *n* = 5. The HS and LS group sizes (*n* = 24 and *n* = 27, respectively) were thus deemed more than sufficient for our hypothesis.

Based on the pilot study, the research and analysis plan for the full study was made publicly accessible on Open Science Framework (OSF: https://osf.io/). Our first preregistration, accessible via OSF (https://osf.io/z95q4), includes the results from our pilot study and outline (i) *a priori* definitions of ROIs and hypotheses based on the pilot results, (ii) rationale for statistical analyses and issues regarding multiple comparisons, and (iii) the relation to our previous preregistration (https://osf.io/tcnw6) of the most central comparisons (ParPrec, ToM and ToM–Lang). The additional analyses involving the language network were preregistered separately during the review process (see §2e).

### (d) Full study (ParPrec, ToM, ToM–Lang, ToM–ParPrec)

Our frequentist analyses were run using the JASP software [40], with an alpha set at 0.05. We performed one-sample *t*-tests on all four network correlations. Based on the tests for group differences run in the pilot, two-sample one- or two-tailed *t*-tests were performed, assuming equal variance between groups. The direction of the two-sample *t*-tests followed the trends of the *t*-statistics from the pilot: HS > LS (higher FC for the HS group) for the ParPrec and ToM–Lang correlations, and LS > HS (higher FC for the LS group) for the ToM–ParPrec correlation. We chose a two-tailed test for the within-network ToM correlation in the full sample, as the *t*-statistic in the preregistered pilot for the current study showed a different trend than our previous preregistration (see §2c).

We report both raw *p*-values and *p*-values using Bonferroni multiple comparison correction for our four group comparisons. A strict statistical threshold for the four comparisons would be 0.05/4 = 0.0125. From a statistically conservative standpoint, raw *p*-values between 0.0125 and 0.05 would be discussed as *nominally* significant. However, as described in our preregistration, it can be argued that correction for multiple comparisons is not needed in this case, as we have stated our four hypotheses *a priori* based on the pilot and are interested in their individual outcomes [41,42]. In addition, multiple comparison correction is only relevant for the first hypothesis, as we expected the null effects of the remaining three to hold in the full sample. Based on this argumentation, the formal label nominal significance may in this case be interpreted as significance.

#### (i) Bayes factor

In addition to frequentist analyses of potential group differences, we performed Bayesian *t*-tests to obtain Bayes factors for both the non-significant and significant results in the full sample. The Bayesian *t*-tests were performed in JASP [40], set as one- or two-tailed following the same rationale as described in §2d. As we set different directions for the tests, we report the results as BF rather than BF_10_, BF_+0_ or BF_-0_. All tests used a default prior scale of √2/2. We interpreted Bayes factors as proposed by Lee & Wagenmakers [43]. For the hypothesized null results, the Bayes factor can provide support for the absence of group differences, rather than solely the lack of an observed effect.

As a final step, we combined the results from the pilot and the full sample in a meta-analytic Bayes factor, using the meta.ttestBF function from the BayesFactor package [44,45] in RStudio [46]. These generally used the same settings as the first Bayesian *t*-tests, unless the trend of the pilot and full sample differed. In these cases, the metaBF was run two-tailed.

### (e) Additional analyses on Lang and Lang–ParPrec

**Figure 3.**
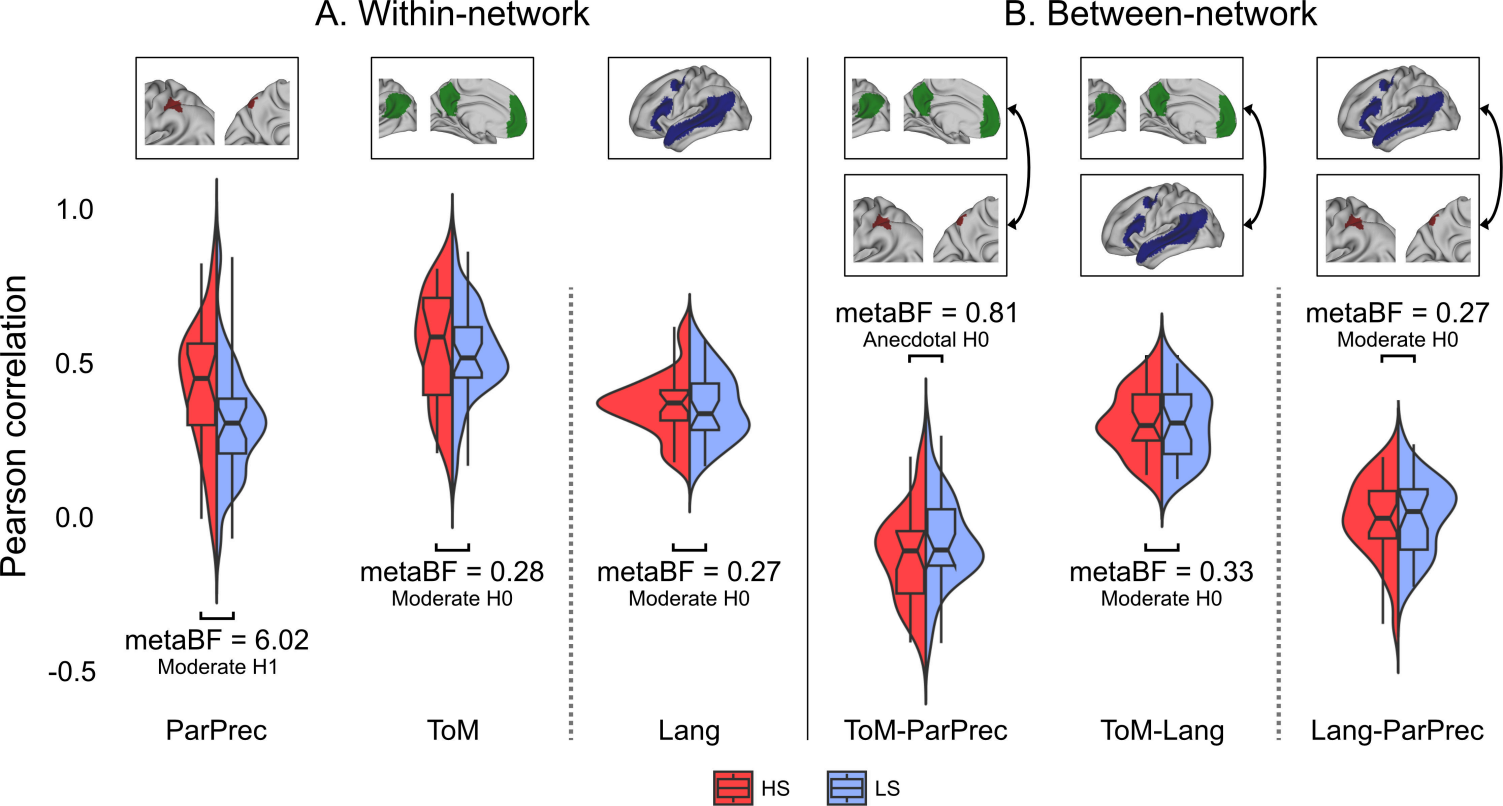
The split violin plots show the distribution of within- and between-network correlations per group. Data include pilot participants. metaBF is a meta-analytical measure, combining the results of the two-sample *t*-tests from the pilot and full sample.

During the review process, we added two further analyses: within-network connectivity of the language network (Lang) and between-network connectivity of the language network and the ParPrec-clusters (Lang–ParPrec). These new analyses followed the same procedure as those described in the previous sections. We created a separate preregistration for these analyses (https://osf.io/6tjns), which includes pilot results of these additional tests. As we preregistered these analyses separately, we treat them as independent questions from the four initial ones. Thus, we do not consider these tests as influencing our previous approach for multiple comparison correction (i.e. we will not correct for six tests, see §2d). To indicate this, they are reported in separate panels in tables 1 and 2 and figure 3.

**Table 1.** Mean functional connectivity across groups in the pilot sample, and results from frequentist and Bayesian two-tailed, two-sample *t*-tests. Negative *t*-statistics indicate higher connectivity in the HS group (HS > LS). Int., interpretation (of Bayes factor); A., anecdotal support.

	mean FC ± s.d. (*n* = 8)	*t*_6_	*p*_raw_	BF (Int.)
ParPrec	0.30 ± 0.13	−2.81	0.031*	2.56 (A. H1)
ToM	0.63 ± 0.17	0.31	0.764	0.54 (A. H0)
ToM–Lang	0.35 ± 0.13	−0.16	0.877	0.53 (A. H0)
ToM–ParPrec	−0.21 ± 0.11	0.73	0.492	0.61 (A. H0)
Lang	0.37 ± 0.09	−0.86	0.424	0.65 (A. H0)
Lang–ParPrec	−0.11 ± 0.09	1.54	0.175	0.97 (A. H0)

**Table 2.** Mean functional connectivity across groups in the full sample and results of one- and two-sample frequentist and Bayesian *t*-tests. metaBF was calculated through combining the *t*-statistics of the pilot and full study. All metaBF tests used the same direction as the corresponding two-sample *t*-tests and used a default prior scale of √2/2. As our two-sample *t*-tests had different directions, BF indicates either BF_10_ (two-tailed), or BF_+0_ or BF_−0_ (one-tailed), indicated by ≠ (two-tailed) or > (one-tailed, testing if higher FC to the left). *P*_corr_ was calculated with Bonferroni correction. FC, functional connectivity; CI, confidence interval; Int., interpretation; M., moderate support; A., anecdotal support.

	FC across groups (HS+LS) (one-sample *t*-tests against 0)				FC between groups (two-sample *t*-tests)				
	mean FC ± s.d. (*n* = 51)	*t*_50_	*p*_raw_	95% CI	test-direction	*t*_49_	*p*_raw_	*p*_corr_	metaBF (Int.)
ParPrec	0.37 ± 0.20	12.99	<0.001*	[0.31, 0.43]	HS > LS	−1.77	0.041*	0.16	6.02 (M. H1)
ToM	0.53 ± 0.15	24.30	<0.001*	[0.48, 0.57]	HS ≠ LS	−0.51	0.611	1	0.28 (M. H0)
ToM–Lang	0.30 ± 0.10	20.32	<0.001*	[0.27, 0.33]	HS >LS	−0.24	0.407	1	0.33 (M. H0)
ToM–ParPrec	−0.09 ± 0.14	−4.28	<0.001*	[−0.12,−0.05]	LS > HS	0.97	0.169	0.68	0.81 (A. H0)
Lang	0.36 ± 0.11	23.81	<0.001*	[0.33, 0.39]	HS > LS	0.20	0.577	1	0.27^a^ (M. H0)
Lang–ParPrec	0.02 ± 0.12	0.99	0.328	[−0.02, 0.05]	LS > HS	−0.28	0.608	1	0.27^a^ (M. H0)

^a^ metaBF was run two-tailed, as the trends from the pilot and full sample showed opposite directions.

From the two-sample, two-tailed *t*-tests of this pilot, we hypothesized that we would see no significant group differences, either within the language network or between Lang–ParPrec.

## 3. Results

### (a) Pilot

Table 1 presents mean functional connectivity across groups and the pilot results. Both frequentist and Bayesian two-tailed, two-sample *t*-tests were performed. A significant group difference was found between the ParPrec-clusters (*t*_6_ = −2.81, *p* = 0.031), with HS (mean = 0.40, s.d. = 0.05) showing higher connectivity than LS (mean = 0.22, s.d. = 0.12). While none of the other comparisons showed significant group differences, all comparisons were included for further tests. As described in §2c,e, and in our preregistrations, we hypothesized that these null results would hold in the full sample, although we used the trends of the *t*-statistics to inform our subsequent tests in the full sample. Detailed descriptive statistics per group are reported in electronic supplementary material, table S1.

### (b) Full sample results

Detailed descriptive statistics of our four main comparisons in the full sample are reported in electronic supplementary material, table S1. In the upper panel of table 2, we report the mean and standard deviations across the two groups, and the results from one-sample and two-sample *t*-tests. For all comparisons except one, the mean correlations were positive both within and between the networks, for both groups. The ToM–ParPrec mean correlations stood out as the only mean correlation with a negative value, although it can be considered weak to negligible in effect size (mean FC −0.09 with 95% confidence intervals from −0.12, −0.05).

In the frequentist two-sample *t*-tests, only the within-network ParPrec comparison reached (nominal) significance (*t_49_* = −1.77, *p*_raw_ = 0.041). The HS group had higher connectivity (mean = 0.42, s.d = 0.22) than the LS group (mean = 0.32, s.d = 0.18). None of the three analyses including the canonical ToM network showed significant group differences (although the trend of within-network ToM connectivity changed polarity compared with the pilot, to HS > LS).

The last column of table 2 combines the results from the pilot and full study in a meta-analytical Bayes factor, here called metaBF. We gathered moderate support (metaBF = 6.02) for a group difference in the case of within-ParPrec connectivity. The other comparisons showed either moderate (ToM and ToM–Lang) or anecdotal (ToM–ParPrec) support for no group difference. Figure 3 plots the distribution of correlations for each group, including the data from the pilot study. The individual BF for each comparison in the full sample can be found in our electronic supplementary material, table S2.

### (c) Additional language network results

The results from our additional tests, treated separately with respect to the Bonferroni correction, are included in the second (lower) panel of table 2. Connectivity within the language network was positive across groups. The mean correlation of Lang–ParPrec was not significantly different from 0.

Neither of the two-sample *t*-tests showed significant group differences, and both tests changed polarity compared with the pilot (Lang to LS > HS, Lang–ParPrec to HS > LS). In the final metaBF, both comparisons showed moderate support for no group difference. The distribution of correlations from these additional tests is included in figure 3. Detailed descriptive statistics and individual BFs for the two comparisons are reported in electronic supplementary material, tables S1 and S2, respectively.

### (d) Follow-up analyses

We report detailed results from a number of follow-up tests in electronic supplementary material, S3.3 and table S3. These tests included the following: (i) corresponding tests in the right hemisphere and (ii) tests on whether removing the overlap between the dorsal precuneal cluster and the posterior cingulate cortex/ventral precuneus ToM ROI had any effect on the ToM–ParPrec comparison, in either hemisphere. The tests showed no substantial differences from the main analyses, and the right hemispheric results support the overall pattern of results reported, as they provide additional (and largely independent) results pointing in the same direction.

## 4. Discussion

We have gathered support for the pragmatic relevance of resting functional connectivity between two clusters previously suggested [14] to be involved in higher level pragmatic processing. The cluster in the left intraparietal sulcus and the bilateral dorsal precuneal cluster (the ‘ParPrec-clusters’) were more closely connected among a group of young adults with higher pragmatic ability (HS), compared with a group with lower abilities (LS). While these groups were created based on individual differences in pragmatic ability, we have demonstrated that this individual pragmatic variability is independent from individual variability in (i) ToM network connectivity, (ii) the connectivity between the ToM and language networks (ToM–Lang), and (iii) how close the pragmatically linked ParPrec-clusters are connected with the ToM network (ToM–ParPrec). The latter result, taken together with the result that the mean between-network connectivity showed that the ToM network and ParPrec-clusters were not connected at all (in any of the groups), clearly suggests that the ParPrec-clusters are not an extension of the ToM network. Considering that we also have gathered Bayesian support for an absence of group differences in connectivity (rather than simply a lack of observed effect) for all our comparisons involving the ToM network, we conclude that there is a partial segregation of ToM and pragmatics.

Along the same lines, we have also shown that the ParPrec-clusters are not an extension of the language network. Thus, we can similarly conclude that language and pragmatics should be considered at least partially segregated. One possible, albeit tentative, interpretation of our results is that the ParPrec-clusters are recruited as a mini-network of two interconnected nodes suitable for high-level pragmatic processing (however, see next paragraph).

### (a) Pragmatic relevance of the ParPrec-clusters

One of the aims of the present investigation was to test whether the activation in the ParPrec-clusters, found in Bendtz *et al.* [14], reflects two separate or a joint process. The significant positive correlation (see one-sample tests, table 2) across groups indicates that these two regions are functionally connected, even at rest. While the mean level of connectivity within the ParPrec-clusters (FC = 0.37) is on a par with e.g. the language network (FC = 0.36), we want to be careful in specifying our interpretation of these nodes as a tentative pragmatic *network*. What the mean level of connectivity within the ParPrec-clusters does suggest is that they regularly exchange information with each other, and that the activity found in Bendtz *et al.* reflects a joint process, related to pragmatics.

As hypothesized based on the pilot result, we also gathered moderate Bayesian support for a group difference (HS > LS) in functional connectivity between the two ParPrec-clusters. While both groups showed positive correlations separately, the higher connectivity for the HS group at rest could partially cause the subsequent increased activation observed during the pragmatic task. Indeed, across seven tasks including language, reasoning and social processing, Cole *et al.* [47] found that individual resting-state connectivity predicted individual differences in cognitive task activation. The underlying neurophysiological reason was suggested to be activity flow as shaped by average synaptic connectivity, which implies that more generally and at larger time scales, repeated co-firing in two areas might also cause Hebbian wiring [48] between these areas (resulting in a coevolution of individual resting-state connectivity and task-based co-activation). Irrespective of whether this is true, our results further strengthen the interpretation that the ParPrec-clusters are involved in pragmatic processing, and that the degree of connectivity is related to pragmatic outcomes, at least those that we measured behaviourally. Note that we do not claim pragmatics to be the *only* function of these regions. The exact process that the activity within the ParPrec-clusters reflects is not yet known. We do, however, consider them to be associated with higher level pragmatics (see some further suggestions based on their locations in §4c). In any case, we have gathered evidence that the clusters should be considered pragmatically *relevant.*

### (b) Segregation of pragmatics and Theory of Mind (as well as language)

The main aim of our study in the context of the current special issue was, however, the relationship between ToM and pragmatics. Our one-sample *t*‐test of functional connectivity between the ToM network and ParPrec-clusters showed a significant, albeit weak, negative correlation in both groups. As we consider the correlation to be negligible, we do not interpret it as a robust negative correlation, but simply a lack of connectivity. Based on the same result, we conclude that the ParPrec-clusters cannot be seen as an extension of the ToM network, especially considering that regions within the ToM network were themselves positively correlated in both groups (see table 2 and electronic supplementary material, S4.1). As we have identified the ParPrec-clusters as being jointly pragmatically relevant, this lack of between-network connectivity suggests a functional segregation of the general ToM network and some aspect of pragmatic processing, and that these two networks do not consistently co-activate or exchange information. Additionally, our pragmatic measures were independent from the degree of connectivity between our pragmatic clusters and ToM, as we found Bayesian support for no differences between the groups for this measure, strengthening the conclusion of a partial segregation.

As already stated in the initial discussion, all three tests of group differences involving ToM support a partial segregation between ToM and pragmatics. This stands in contrast to accounts that situate pragmatics within a larger ToM construct, such as it being a sub-module [2]. If the latter was true, there should be some relationship between measures of functional connectivity of ToM and pragmatic neural or behavioural measures. As none were found, our results go in line with what was reported in Bendtz *et al.* [14]: a lack of correlation between the pragmatic and ToM behavioural tasks and activity in the ParPrec-clusters and the ToM task (electronic supplementary material, figure S1), all supporting a partial segregation.

We do not deny that ToM does play a role in pragmatic processing and we consider our proposal of a segregation as truly partial, not full. Activation within the ToM network has consistently been observed during pragmatic tasks similar to ours [14–16], and was also found in the meta-analysis by Hauptman *et al.* [19], which included a wide range of pragmatic phenomena. However, as has been shown in both neuroimaging [18] and behavioural [11] studies, ToM is not always engaged or associated with pragmatic processing or performance. This already indicates some level of segregation between the two, although the exact reason for whether ToM is required is not known. We develop a related proposal in section §4c.

Our present conclusion that ToM and pragmatics can be at least partially segregated is based on our findings that individual differences in pragmatic ability cannot be explained by functional connectivity related to the ToM network. This is supported by our main results of Bayesian support for an absence of group differences (within ToM and between ToM–ParPrec) as well as a more general lack of connectivity between the ToM network and the ParPrec-clusters.

Similar to the ToM network, the language network has also been shown to be engaged during pragmatic processing, and the two networks correlate above baseline [23]. The fact that we found no group differences in ToM-related connectivity poses the question of if, instead, language, or a combination of ToM and language, could be a substantial source of individual pragmatic variability. In Bendtz *et al.* [14], we also tested for group effects of the contrast ‘language processing’ versus ‘silence’ and found none. This suggests that the groups do not differ on a general level of language processing or attention. To come back to Paunov *et al.* [23], we have here replicated a connection between the ToM and language networks (table 2 and electronic supplementary material, S4.1). However, individual differences in pragmatic skill were not associated with between-network connectivity. Our additional Bayesian tests within the language network and between language and the ParPrec-clusters similarly showed support for independence of connectivity and pragmatic variability. As with the ToM network, we can therefore also conclude that the language network is not connected to the ParPrec-clusters. Thus, our results show not only that pragmatics can be partially segregated from ToM but also from the language network, as well as the ToM and language networks when regarded as two networks working in tandem.

### (c) Revisiting neuropragmatics in the context of large-scale functional networks

The observed segregation is of course limited to the three aspects of pragmatics that the current study is based on: (i) audience design and (ii) pragmatic prosodic processing as the basis of the group division (HS and LS), and (iii) the localization of the ParPrec-clusters as a function of group differences (HS > LS) in indirect speech act processing in Bendtz *et al.* [14]. But what, beyond the already reviewed empirical evidence, might be gauged based on the neuroimaging approach to pragmatics more generally, regarding such a segregation? And if pragmatics is not a submodule of ToM and neither the combination of ToM and structural language, how can it be described or reconstructed? While we cannot offer more than an inkling, we want to start by noting that the mere time modern humans spend in conversation is substantial. In analogy, face processing is similarly central to being human, and a face processing region has evolved, classically located in the human ventral visual ‘what’ processing stream (we note, however, that in recent accounts, there is a third visual processing stream dedicated to socially relevant visual cues [49,50]). These observations, together with inspiration from Levinson’s interaction engine [51], lead us to propose that the bulk of pragmatics may be covered in an expert domain, building on, among others, ToM and language (as well as cognitive control processes, see §4d), but specific to the conversational context. We are at least imagining a future reconstruction of pragmatics based on similar neurocognitive and evolutionary considerations.

We will now revisit (i) studies of two posterior regions (the ParPrec-clusters in this article) and (ii) studies of the medial prefrontal cortex, as this area is a key node in ToM, social cognition as well as studies on pragmatics. Starting with (i), a neurocognitive proposal of an expert system for pragmatic inferential processing must build on some anatomical segregation from the ToM and language networks, which is what we established for the ParPrec-clusters. Underlying representations in such potential expert areas may include abstract situation model representations in the superior parietal lobe [52] and potentially the dorsal precuneus. The more dorsal aspect of the precuneus has been shown to be specialized for person representations, compared with more ventral place representations (see [53], but also [54], where time/event areas are also included).

As for (ii), more anteriorly, comprehension of indirect speech acts activates the superior portion of the midline prefrontal cortices [16] or the superior and more anterior portion [14]. When considering pragmatic production, as in a study on audience design [55], activity in a more ventral portion of the posterior midline prefrontal cortex was observed. What is crucial to note is that some of these medial prefrontal activations are posterior to midline anterior prefrontal ToM areas, thus again partially segregating pragmatics from ToM (see the location of conversational production activity when uttering less predictable words in [56]; electronic supplementary material, figure S2). Our proposal that pragmatic (more posterior) medial prefrontal activations are driven by specialization to the conversational context is consistent with the general pattern of abstract processes as represented more anteriorly and more context-specific processes as represented more posteriorly, within an anterior–posterior gradient in the frontal lobe [57,58]. We also propose a consistent developmental perspective, where indirect speech acts start to be processed by the more abstract domain-general ToM system, based on our own data from 51 adolescents ([22]; see electronic supplementary material, figure S3) performing the same indirect speech act task as in Bendtz *et al.* [14]. Along with an expert system developing, processing of indirect speech acts may recruit posterior areas ([14,22]; see electronic supplementary material, figure S4).

Here, we have outlined the conversational context as crucial for specialization and segregation of pragmatics from domain-general (and more abstract) ToM. However, our proposal also bears resemblance to how *communicative situations* are considered by Katsos & Andrés-Roqueta [5], and it can be modified to construe context-specificity for pragmatics in other ways (e.g. specificity to particular interlocutors or social situations). We have proposed that the ParPrec-clusters, as well as medial prefrontal regions posterior to prefrontal ToM areas, are three locations that may harbour such representations. It is, however, important to again note that other neural processes implementing various pragmatic phenomena or situations also recruit other areas, including ToM and language areas [59] (see also electronic supplementary material, figure S4). In addition, we are not suggesting that the mentioned three locations are exclusively dedicated to pragmatic processing.

### (d) Additional brain networks and future directions

While we have shown a partial segregation between pragmatics and ToM, the neuroimaging approach has hitherto failed to clearly segregate pragmatics from executive functions/cognitive control functions as represented in the multiple demand (MD) network ([60], see, however, [19]). Our own preliminary pilot results suggest a high degree of connectivity between the parietal cluster and the MD network (mean FC = 0.40) but no connectivity with the dorsal precuneal cluster (FC = 0.06). Furthermore, both clusters overlap with a lateral dorsal posterior system described as supporting intuitive physical reasoning [61], or physical inferencing [62]. This system is itself partially overlapping with the general MD network, though it is, at least in part, dissociable from it [61]. Additionally, the default mode network (DMN) should be considered, especially as there is anatomical proximity of the ParPrec-clusters and regions often considered part of the DMN (see further comparison in electronic supplementary material, S4.3). We suggest that follow-up studies perform similar in-depth resting-state analysis, as in the current study, but instead with respect to these other large-scale networks.

## 5. Conclusion

We interpret our results as indicating that aspects of pragmatics are distinct from ToM and language, and that pragmatics should be considered partially segregated from the two domains. While both ToM and language have some role in pragmatic processing, we show that neither functional connectivity within the ToM or language networks, nor between ToM and language, explains variability in pragmatic skill. Moreover, we have strengthened our earlier finding that the ParPrec-clusters, located outside both the language and ToM networks, are relevant for pragmatic behavioural outcomes. The two clusters were robustly connected, and higher resting-state connectivity was associated with higher scores on two other pragmatic tasks. Furthermore, the observed lack of connectivity between these clusters and the general ToM and language networks cannot be accounted for in case of no segregation. In conclusion, pragmatics should not be considered a subset of ToM, an extension of the language network or as a joint process of ToM and language.

## Data Availability

The data supporting this article have been uploaded as part of the supplementary material. Supplementary material is available online [63].
